# The Management Dilemma: Concomitant Acute Hip Fracture and Severe Asymptomatic Aortic Stenosis

**DOI:** 10.7759/cureus.7527

**Published:** 2020-04-03

**Authors:** Talha Ahmed, Ayesha Safdar

**Affiliations:** 1 Internal Medicine, University of Maryland Medical Center, Baltimore, USA; 2 Internal Medicine, Army Medical College, Rawalpindi, PAK

**Keywords:** aortic stenosis, hip fracture, transcatheter aortic valve replacement, management rationale, elderly patients, surgical hip repair

## Abstract

Acute hip fractures (AHF) are common in elderly patients. A combination of age-related osteoporosis and increased fall risk makes this population group most susceptible to different fractures including acute fracture of the hip. AHF is a disabling condition that warrants immediate attention. It has a huge impact on the already compromised baseline functional status of elderly patients rendering them more susceptible to different morbidities and even mortality. Similarly, age-related degeneration of the aortic valve with resulting calcification also makes elderly patients prone to aortic stenosis (AS). Severe asymptomatic AS when diagnosed in these patients with AHF in the perioperative period makes the management options very challenging. Severity of AS usually translates into worse postoperative outcomes. The management rationale of concomitant presence of these two conditions is unclear. There is a lack of clear-cut recommendations and societal guidelines in such scenario.

## Introduction and background

A major health concern in elderly patient population is acute hip fracture (AHF). As the number of elderly patient population is rising, the prevalence of hip fractures is also on a rise. Female gender is most commonly affected by this condition [1]. Other factors like age, comorbidities, anticoagulation therapy, and general physical health status at the time of injury also determine the prognosis of these patients. Hip fractures have the propensity of being fatal if not adequately addressed. Mortality rates among the elderly following hip fractures range between 14% and 36% within one year of the injury. During the first three months after hip fracture, elderly patients have a fivefold to eightfold increased risk of dying. The increased mortality risk persists up to 10 years [2].

Calcific aortic stenosis (AS) is common in elderly patients due to age-related valve degeneration and calcification. Symptomatic severe AS mandates valve replacement. Due to the prevalent use of echocardiography, more elderly patients are now being diagnosed with asymptomatic severe AS. The current standard of care involves considering valve replacement in these individuals especially those who are at an increased risk of developing complications [3].

A particularly challenging situation arises when a patient who comes with AHF is diagnosed with severe AS on preoperative evaluation. The exact management rationale in this scenario is not clear. Different treatment options exist for each of these diagnoses, and the treatment is guided by patients’ presenting symptoms and clinical expertise of the physician. It usually involves shared decision making between surgeon, cardiologist, and the patient [4,5]. The purpose of this review is to highlight the current literature available in these scenarios to help guide physicians about the current standard of care for these patients.

## Review

The incidence of osteoporotic fracture increases exponentially throughout life, as does the risk of the devastating consequences of these fractures. These include but are not limited to functional decline, institutionalization, immobilization, elderly neglect, and even mortality. AHF is a debilitating injury that impacts the duration and quality of life of elderly (particularly in elderly > 65 years of age). Females are more commonly affected than males [6]. Aside from gender, data support that patient demographics play a role in hip fractures. Results from a study revealed that Caucasians had the highest incidence of hip fractures across all race/ethnicities, whereas Native Americans had the lowest rates. The American Academy of Orthopaedic Surgeons' evidence-based clinical practice guideline (2016) on hip fracture diagnosis and treatment in geriatric patients stated that moderate evidence supports that hip fracture surgery within 48 hours of admission is associated with better outcomes. However, delaying surgery may be necessary to stabilize patients with significant comorbidities and obtain preoperative medical clearance [7].

Another prevalent diagnosis in geriatric patients is age-related AS. It is basically a degenerative valvular heart disease, which represents the most common etiology of AS in the elderly population. Degenerative aortic valve disease affects over 25% of all patients over the age of 65 years. Most patients have only mild thickening and normal valve function, called aortic sclerosis. However, 2%-5% of these patients have significant AS with obstruction of left ventricular outflow [8,9].

Various treatment options for AHF include close reduction and percutaneous pinning (usually done in very frail patients with multiple comorbidities), intramedullary nailing, arthroplasty (hemiarthroplasty and total arthroplasty), and open reduction and internal fixation (ORIF). The choice of the treatment relies on extent of fracture, burden of comorbidities, baseline functional status of patient, clinical presentation, institutional practice, and physician expertise. AS management options include balloon valvuloplasty (temporary opening of aortic valve with balloon without valve replacement to allow transient improvement in stenosis), transcatheter aortic valve replacement preferred in old patients when surgical risks are prohibitive, and surgical aortic valve replacement preferred in relatively young patients with low surgical risks and in certain cases of congenital aortic valve stenosis (due to longevity of the surgically placed valve in these young patients). However, when these two conditions (AS and AHF) are present simultaneously in a patient, the management becomes challenging. Delaying surgery for AHF increases the morbidity and mortality and reduces the chance of complete healing. At the same time, the presence of AS increases risk of anesthesia-related complications as well as perioperative cardiac complications (hypotension requiring vasopressors and inotropes, worsening heart failure, arrhythmias, myocardial infarction, and cardiogenic shock) [10,11].

In an attempt to analyze this issue in elderly, Leibowitz et al. conducted a case-control study in 2009 to evaluate the effect of severe AS in patients with surgically repaired hip fracture. Patients with an echocardiographic diagnosis of severe AS (n=33 with median age of 84.5 years) were age-matched with patients without a history of AS (n=88 with median age of 85 years). Both groups underwent surgical repair of AHF. There were no significant differences between the AS group and controls for 30-day mortality (6.2 vs. 6.8%) or for the total cardiac event rate (18.7 vs. 11.8%). The results of this study concluded that there was no short-term difference in outcomes for surgical hip fracture repair regardless of presence or absence of severe asymptomatic AS [12].

Keswani et al. in 2016 conducted a similar retrospective case-controlled review (1:2 age-matched groups) of elderly (≥65 years) surgically treated hip fractures from 2011 to 2015 with moderate to severe AS (according to American Heart Association criteria). A total of 65 cases were compared to 129 controls. Postoperative complication rates, 30-day and one-year mortality were reviewed. The hip fracture treatment in case vs control group was closed reduction and percutaneous pinning 5 (7.7%) vs. 16 (12%), hemiarthroplasty 13 (20%) vs. 28 (22%), intramedullary nail 26 (40%) vs. 45 (35%), and ORIF 21 (32%) vs. 40 (31%). Of note, patients who underwent an aortic replacement procedure within last 12 months before and after the study were excluded [13]. Hence, these patients, similar to those studied by Leibowitz et al., had untreated moderate to severe asymptomatic AS [12]. However, unlike the results of that study, the outcomes were worse for the patients included this study. These results reported by Keswani et al. urged the clinicians to be more vigilant in their management of moderate to severe asymptomatic AS in patients undergoing hip fracture surgery [13]. 

A recent (2019) case-control study was done by Rostagno et al. involving 145 patients with AS and 283 consecutive patients without AS (control group) aged >70 years with hip fracture [14]. A total of 66 patients had mild AS, 47 had moderate AS, and 32 had severe AS according to the European Society of Cardiology guidelines. Thirty-day mortality was 6.2% in AS and 3.1% in controls. Postoperative non-fatal myocardial infarction and composite endpoint were more frequent in AS than in the control group (8.3% vs. 1.1%, p<0.001 and 14.5% vs. 4.2%, p<0.001, respectively). The risk was significantly higher for patients with severe AS (28.1%). One-year mortality in patients with moderate to severe AS was 46% in comparison with 16% in those with mild AS or in the control group (p<0.001). Coronary disease, atrial fibrillation, age, and aortic gradient were independent predictors of mortality in AS [14] . A preoperative diagnosis of severe AS changed the approach to management of these patients. For patients with severe AS, surgery was performed under general anesthesia in 90% cases and arterial line was positioned for continuous hemodynamic monitoring. More than half of patients were observed overnight in intensive care unit (ICU) for hemodynamic monitoring. Only 5% required longer ICU for hemodynamic instability. The results of this study concluded that involvement of dedicated cardiac team to evaluate the need for perioperative AS management may help improve outcomes in these patients.

This review of literature shows that there is more evidence in favor of worse outcomes if the AS is left untreated in patients with planned AHF repair. These include perioperative, 30-day, and one-year outcomes. With the improvement in technique of catheter-based procedures including valve replacement and valvuloplasty, the perioperative management of these patients can be more optimized to improve their outcomes. It has always been a management dilemma when a patient presents with AHF and is found to have a severe AS on echocardiogram. There is a lack of clear-cut guidelines and societal recommendations. Shared decision-making and expert opinions help guide management in such situations. 

## Conclusions

Elderly patients represent a vulnerable group with increased burden of age-related comorbidities. AHF in geriatric population, resulting from weak bones (osteoporosis) and susceptibility to falls (balance issues), is a debilitating condition that warrants immediate medical attention. A preoperative diagnosis of asymptomatic severe AS makes the situation complicated in such scenarios. A few years back, the usual practice was to proceed with surgery of hip repair and optimize the perioperative condition of the patient with close monitoring. With advancements in hip repair strategies and the widespread use of catheter-based procedure in patients with AS, we now have more options to address this dilemma. There is a need for dedicated prospective study to address this concern. Meanwhile, individual patient scenarios can help guide the treatment rationale after shared decision-making between the patient, surgeon, anesthesiologist, and the cardiologist to lessen the harm and provide optimal care to this growing population stratum in United States. 

## References

[1] Pincus D, Ravi B, Wasserstein D (2017). Association between wait time and 30-day mortality in adults undergoing hip fracture surgery. JAMA.

[2] Zingmond DS, Melton LJ III, Silverman SL (2004). Increasing hip fracture incidence in California Hispanics, 1983 to 2000. Osteoporos Int.

[3] Finn M, Green P (2014). Transcatheter aortic valve implantation in the elderly: who to refer?. Prog Cardiovasc Dis.

[4] van de Ree CLP, De Jongh MAC, Peeters CMM, de Munter L, Roukema JA, Gosens T (2017). Hip fractures in elderly people: surgery or no surgery? A systematic review and meta-analysis. Geriatr Orthop Surg Rehabil.

[5] Roberts KC, Brox WT (2015). AAOS clinical practice guideline: management of hip fractures in the elderly. J Am Acad Orthop Surg.

[6] Pedersen AB, Ehrenstein V, Szépligeti SK, Sørensen HT (2017). Hip fracture, comorbidity, and the risk of myocardial infarction and stroke: a Danish Nationwide Cohort Study, 1995-2015. J Bone Miner Res.

[7] Anthony CA, Duchman KR, Bedard NA (2017). Hip fractures: appropriate timing to operative intervention. J Arthroplasty.

[8] Iung B, Vahanian A (2011). Epidemiology of valvular heart disease in the adult. Nat Rev Cardiol.

[9] Eveborn GW, Schirmer H, Heggelund G, Lunde P, Rasmussen K (2013). The evolving epidemiology of valvular aortic stenosis. the Tromsø study. Heart.

[10] Martinsson A, Li X, Andersson C, Nilsson J, Smith JG, Sundquist K (2015). Temporal trends in the incidence and prognosis of aortic stenosis: a nationwide study of the Swedish population. Circulation.

[11] Arnold SV, Afilalo J, Spertus JA (2016). Prediction of poor outcome after transcatheter aortic valve replacement. J Am Coll Cardiol.

[12] Leibowitz D, Rivkin G, Schiffman J (2009). Effect of severe aortic stenosis on the outcome in elderly patients undergoing repair of hip fracture. Gerontology.

[13] Keswani A, Lovy A, Khalid M (2016). The effect of aortic stenosis on elderly hip fracture outcomes: a case control study. Injury.

[14] Rostagno C, Ranalli C, Polidori G, Cartei A, Boccaccini A, Peris A (2019). Outcome in elderly patients with aortic stenosis undergoing hip fracture surgery. Results may suggest a different postoperative strategy?. Trauma Surg Acute Care Open.

